# Solution-Processed Cu_2_ZnSn(S,Se)_4_ Thin-Film Solar Cells Using Elemental Cu, Zn, Sn, S, and Se Powders as Source

**DOI:** 10.1186/s11671-015-1045-6

**Published:** 2015-08-21

**Authors:** Jing Guo, Yingli Pei, Zhengji Zhou, Wenhui Zhou, Dongxing Kou, Sixin Wu

**Affiliations:** The Key Laboratory for Special Functional Materials of MOE, Henan University, Kaifeng, 475004 China

**Keywords:** CZTSSe, Thin film, Solar cells, Kesterite, Solution process

## Abstract

Solution-processed approach for the deposition of Cu_2_ZnSn (S,Se)_4_ (CZTSSe) absorbing layer offers a route for fabricating thin film solar cell that is appealing because of simplified and low-cost manufacturing, large-area coverage, and better compatibility with flexible substrates. In this work, we present a simple solution-based approach for simultaneously dissolving the low-cost elemental Cu, Zn, Sn, S, and Se powder, forming a homogeneous CZTSSe precursor solution in a short time. Dense and compact kesterite CZTSSe thin film with high crystallinity and uniform composition was obtained by selenizing the low-temperature annealed spin-coated precursor film. Standard CZTSSe thin film solar cell based on the selenized CZTSSe thin film was fabricated and an efficiency of 6.4 % was achieved.

## Background

Quaternary semiconductor Cu_2_ZnSnS_4_ (CZTS) and Cu_2_ZnSnSe_4_ (CZTSe) compounds have received considerable interest as new generations of photovoltaic absorbing materials, due primarily to their suitable band gaps, high absorption coefficient, and low material cost [1]. Recently, various approaches have been developed to fabricate the absorber layers, briefly including vacuum-based deposition and non-vacuum-based solution process; both strategies have yielded a remarkable improvement in photovoltaic performance [2–8]. Compared to vacuum-based approaches, non-vacuum technologies such as electrodeposition approach [9–11], milling dispersion approach [12], nanoparticle-based approach [13–16], hydrazine-based approach [17–19], and sol–gel approach [20–24] are more feasible for industrial production. Among those solution-based process, the hydrazine-based deposition has made the great progress, achieving the power conversion efficiency (PCE) of 12.6 % [25]. However, as a result of the high toxicity and dangerous instability of explosible hydrazine, the non-hydrazine solvent is more desirable for practical application. Therefore, some non-hydrazine solvents, such as the mixtures of ethanol and water and amine-thiol mixture, have been tried presently to dissolve the metallic oxide or metal salt for preparing the CZTS precursor solution [26–28].

For CZTS (e) thin film solar cell, it is very crucial to precisely control the elemental composition of quaternary compounds, which regulate the band gap of the semiconductor and further dominate the device performance. From this point of view, the ideal precursor is prepared using elemental Cu, Zn, Sn, and S (e) rather than metallic oxide or metal salt to avoid involving impurity. Furthermore, the elementary metal powder are substantially inexpensive and easy industrially available. Lately, the Pan’s research group has firstly reported an approach to fabricate CZTSe films which used the mixture of thioglycolic acid and ethanolamine to dissolve the Cu, Zn, Sn, and Se powder [29]. In order to adjust the viscosity of the solution for subsequent spin coating, another organic solvent of 2-methoxyethanol was added into the mixed solution.

In this paper, we presented a more convenient and quicker method to fabricate the high-quality CZTSSe thin film. 1,2-ethanedithiol and 1,2-ethylenediamine were adopted as a facile and low-toxic solution to dissolve the low-cost Cu, Zn, Sn, S, and Se powders as starting materials. A solar cell efficiency of 6.4 % was obtained using this novel solution deposition and process procedure of CZTSSe active layer.

## Methods

### Materials

Cu (99.9 %, Aladdin), Zn (99.9 %, Aladdin), Sn (99.8 %, Alfa Aesar), Se (99 %, Alfa Aesar), and S (99.9 %, Aladdin) powders are analytical reagents. 1,2-ethanedithiol (HSCH_2_CH_2_SH, AR), 1,2-ethylenediamine (H_2_NCH_2_CH_2_NH_2_,AR), ammonium hydroxide (NH_4_OH, 25 %), cadmium sulfate (AR), and thiourea (AR) were purchased from Alfa Aesar. All chemicals and solvents were commercially available and used as received without further purification.

### Preparation of CZTSSe Precursor Solution

Cu (1.10 mmol), Zn (0.76 mmol), Sn (0.62 mmol), S (1.50 mmol), and Se (1.50 mmol) were added into a 25-ml round flask. Then, 0.5 ml of 1,2-ethanedithiol and 5 ml of 1,2-ethylenediamine were injected into the flask. The mixture was magnetically stirred on a 70 °C hot plate for 1.5 h and a clear orange-colored solution was obtained.

### Fabrication of CZTSSe Thin Film and Solar Cell Device

The CZTSSe precursor solution was spin-coated on a 2 × 2 cm molybdenum-sputtered soda lime glass (SLG) substrate, followed by heating at 310 °C on a hot plate in argon atmosphere. This coating and sintering procedure was repeated several times till the desired film with thickness of 1.6 μm was obtained. Finally, the thin film was annealed at 550 °C in a graphite box containing 200 mg of Se powder for 15 min. CZTSSe thin film solar cells were fabricated using the selenized CZTSSe thin films by successively depositing the following additional layers: chemical bath deposition (CBD) of ∼60 nm cadmium sulfide (CdS), sputtering of ∼70 nm intrinsic zinc oxide (ZnO), and ∼200 nm indium-doped tin oxide (ITO). On the top of the device, Al collection grid electrodes were deposited by thermal evaporation. No anti-reflection coating was utilized.

### Characterizations

Thermogravimetric analysis (TGA) was performed by a TGA/SDTA851e of Mettler-Toledo. The powder X-ray diffraction (XRD) patterns were taken with a Bruker D8 Advance X-ray diffractometer. The Raman spectra were measured by a Renishaw in via Raman microscope using an excitation laser with a wavelength of 532 nm. The scanning electron microscope (SEM) images were collected using a Nova NanoSEM 450. Photocurrent density-voltage curves were recorded under the standard AM1.5 illumination (100 mW · cm^−2^) with a Keithley 2400 source meter. The external quantum efficiency (EQE) spectrum was measured using a Zolix SCS100 QE system equipped with a 150-W xenon light source and a lock-in amplifier.

## Results and Discussion

**Fig. 1:**
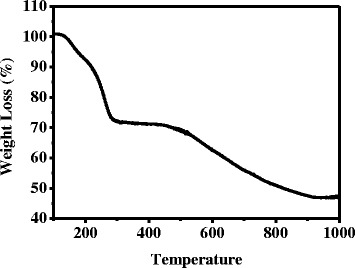
TGA curve of CZTSSe precursor with the target ratios (Cu/(Zn + Sn) = 0.8 and Zn/Sn = 1.22)

Figure 1 presents the thermogravimetric analysis (TGA) curve for the mixed CZTSSe precursor (Cu/(Zn + Sn) ≈ 0.80 and Zn/Sn ≈ 1.22). The TGA sample was prepared from the CZTSSe precursor solution by preheating at 100 °C for 20 min under N_2_ atmosphere to remove the low-boiling-point molecules. It was found that the CZTSSe precursor exhibited a relatively fast weight loss between 130 and 300 °C, which may be ascribed that the extensive N − H · · · S hydrogen bonding existed in the precursor solution was broken and the CZTSSe nanocrystal formed at this temperature [30]. Thereby, in our experiment, CZTSSe nanocrystal thin films were fabricated by spin coating of CZTSSe precursor solution, followed by heating on a hot plate at 310 °C for 5 min. It is noteworthy that in order to form a dense CZTSSe absorbing film, the preheating procedure for removing the organic solvents after each spin coating was essential.

**Fig. 2:**
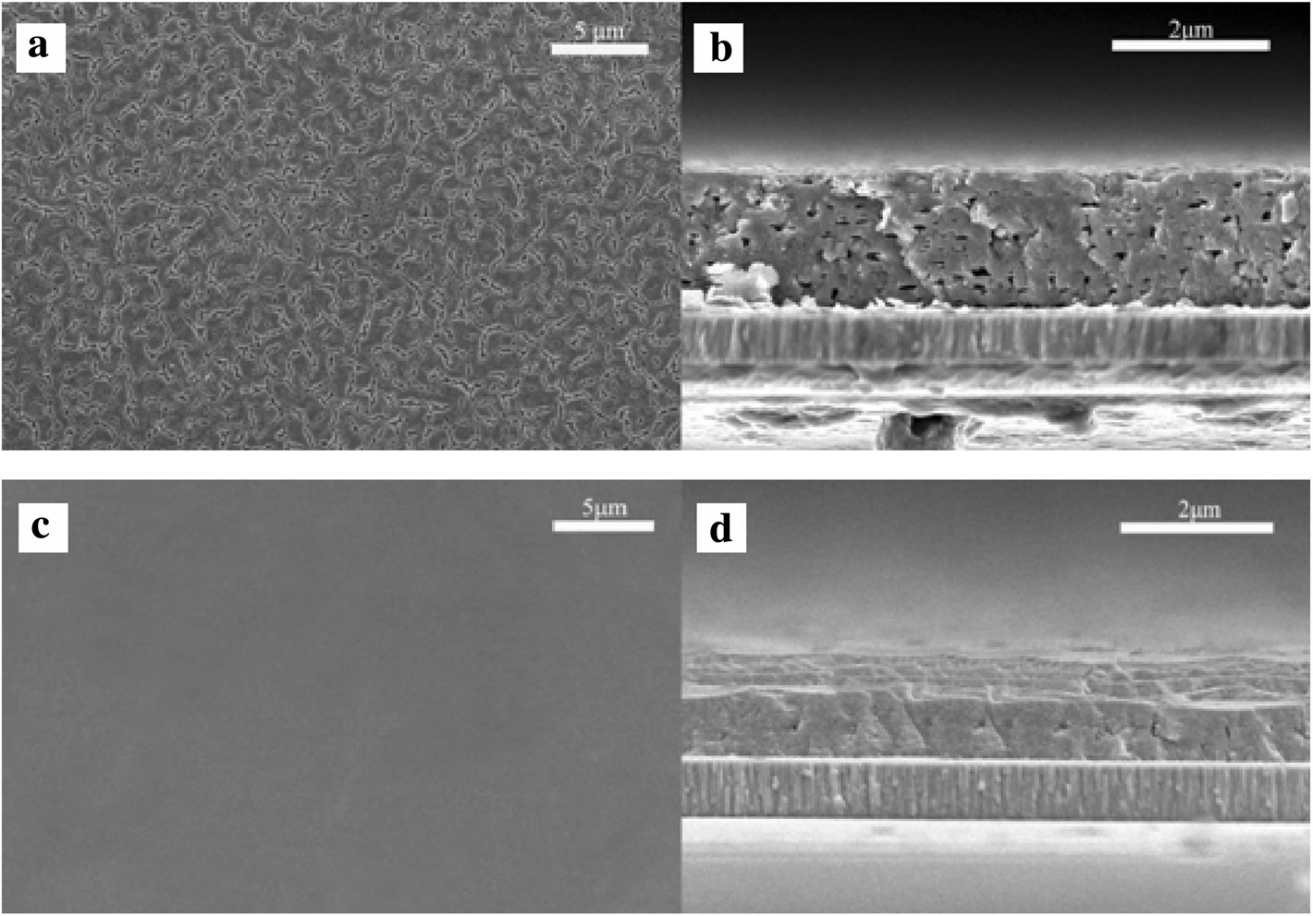
*Top-view* (**a**) and *cross-sectional* (**b**) FESEM images of as-prepared CZTSSe thin film, corresponding *top-view* (**c**) and *cross-sectional* (**d**) FESEM images of CZTS thin film prepared by using S to replace Se atom

The representative surface topography and cross-sectional FESEM images of the as-prepared CZTSSe films are displayed in Fig. 2a and 2b, respectively. From which we can see that a flat thin film with a thickness of 1.6 μm was obtained through repeating the coating/sintering procedure. It is obvious that the CZTSSe nanocrystal thin film was not dense and there were many holes appearing in the film, which may be resulted from the volatilization of partly unreacted Se powder. To confirm this hypothesis, using S to replace Se, the control experiment was done to fabricate CZTS thin film. The corresponding FESEM images are shown in Fig. 2c and 2d. It can be seen that the CZTS nanocrystal films are more compact and dense compared with the CZTSSe films, which were fabricated by the same solution process. Generally speaking, it is undesirable for the existence of pore in the absorbing layer of thin film solar cell, however, the porous structure was beneficial to form well crystallization and large grain CZTSSe thin films in the following selenization process [31].

**Fig. 3:**
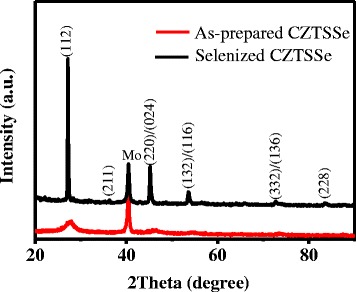
XRD patterns of as-prepared CZTSSe and selenized CZTSSe thin films

To further investigate the phase composition and phase structure of as-prepared CZTSSe film and selenized CZTSSe film, XRD measurements were carried out. Figure 3 displays the X-ray diffraction patterns of the CZTSSe film before and after selenization treatment. Eliminating the peaks arising from Mo substrate, all the diffraction peaks in XRD patterns are attributed to the kesterite phase of CZTSSe (JCPDS no. 52−0868). Comparing with the samples of preheated CZTSSe, the (112) lattice plane of selenized CZTSSe films drifts to lower degree, indicating that partial S in the CZTSSe precursor film was replaced by Se after selenization. In addition to the shifting of diffraction peaks in XRD patterns, the intensity of the diffraction peaks undergoes a significant increase, which demonstrates that the crystallization of the CZTSSe films was dramatically improved after selenization.

**Fig. 4:**
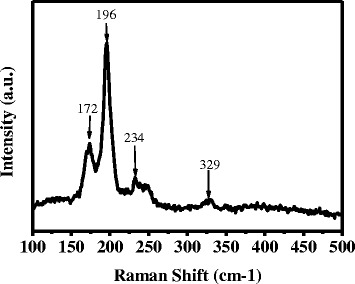
Raman spectrum of selenized CZTSSe thin film

The XRD pattern of some binary and ternary selenide, such as Cu_*x*_Se, ZnSe, and Cu_2_SnSe_3_, are similar to that of CZTSSe, so it is hard to identify the phase purity of CZTSSe films just by XRD characterization. The Raman spectra can be used as an effective measurement to differentiate these impurities. Raman spectra of the selenized CZTSSe film is shown in Fig. 4. The peaks at 176, 192, 234, and 329 cm^−1^ correspond to kesterite CZTSSe [32].

**Fig. 5:**
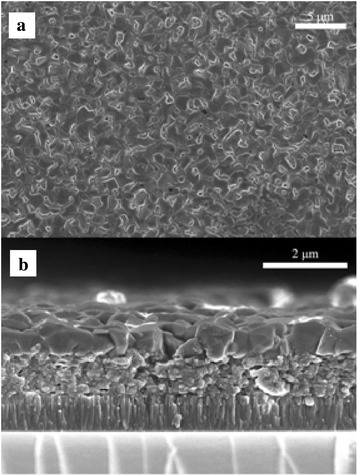
*Top-view* (**a**) and *cross-sectional* (**b**) SEM images of selenized CZTSSe thin film

For kesterite thin film solar cells, the morphology and crystal size of the absorber layer play an important role in the finally photovoltaic performance of the devices. In order to eliminate holes and induce crystallization and large grain formation, the selenization was performed at high temperatures under saturated selenium atmosphere in a graphite box. The morphologies of the selenized CZTSSe thin films were shown in Fig. 5. It was observed that large-grained and densely packed CZTSe thin film with a rough, highly faceted surface has achieved; the average in-plane grain size is 0.5–2 μm, which illustrated that the film converted into a high-crystallinity CZTSSe thin film after selenization. From the fractured cross-sectional view of Fig. 5b, the selenized CZTSSe thin film exhibits a typical bilayer (large-grain layer and fine-grain layer) structure with a thickness of 1.64 μm. Both the thickness of large-grain layer on the top and the fine-grain layer near the molybdenum substrate is about 800 nm.

**Fig. 6:**
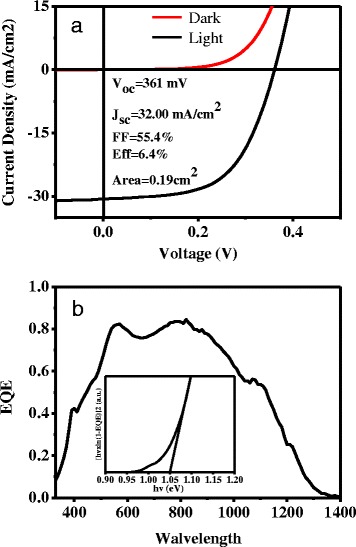
**a** *J*–*V* curves of the best CZTSSe solar cell in the dark and under simulated solar light (AM 1.5 G) illumination. **b** EQE spectrum of the corresponding device; inset: the band gap was determined by plotting [E × ln(1 − EQE)]^2^ versus E curves

Using the selenized CZTSSe thin films, solar cell devices were fabricated through the general substrate-type configuration (Mo/CZTS/CdS/i-ZnO/n-ZnO/Al). The current density–voltage (*J*–*V*) curves of the best CZTSSe solar cell in dark and under AM1.5 illumination are shown in Fig. 6a. The champion device based on an active area of 0.19 cm^2^ yielded a PCE of 6.4 % with short circuit current density (*J*_SC_) of 32 mA cm^−2^, open circuit voltage (*V*_OC_) of 361 mV, and fill factor (FF) of 0.554. For further investigation of the device performance, EQE spectra of the corresponding solar cell were measured and shown in Fig. 6b. The results revealed that the photoresponse of the CZTSSe device was stronger in the visible and near-infrared (NIR) region, which is corresponding well with other high-efficiency CZTSSe solar cell in the literature [29]. The inset of Fig. 6b shows the band gap of CZTSSe thin film by plotting [Eln(1 − EQE)]^2^ versus the photon energy (E), which was estimated to be about 1.05 eV.

Compared with hydrazine-based solution method for CZTSSe thin film, the efficiency of photovoltaic device present in our study is still limited, which may be mainly attributed to the existence of small-grained bottom layer in the selenized CZTSSe thin films. It has been reported that a thick small-grained bottom layer would increase the series resistance of devices and further degrade the photoelectric conversion efficiency of CZTSSe solar cells [33]. Therefore, in order to further improve the performance of solar cells, it is necessary to optimize the selenization conditions for reducing or even completely eliminating the small-grained bottom layer of CZTSe films, which is the ongoing research in our laboratory.

## Conclusions

In summary, a reproducible and lower-toxicity solution-based process for the fabrication of the CZTSSe absorber layer, involving simultaneous dissolution of elemental Cu, Zn, Sn, S, and Se powders in the mixed 1,2-ethanedithiol and 1,2-ethylenediamine solution followed by deposition of a precursor solution, has been presented. After the preheating and post-selenization processes, an extremely dense and compact CZTSSe thin film with high crystallinity has formed. The CZTSSe thin film solar cells fabricated using this process exhibits an efficiency of 6.4 %, which is expected to further enhance by optimizing the composition and selenization of the CZTSSe film.
